# G9a-mediated methylation of ERα links the PHF20/MOF histone acetyltransferase complex to hormonal gene expression

**DOI:** 10.1038/ncomms10810

**Published:** 2016-03-10

**Authors:** Xi Zhang, Danni Peng, Yuanxin Xi, Chao Yuan, Cari A. Sagum, Brianna J. Klein, Kaori Tanaka, Hong Wen, Tatiana G. Kutateladze, Wei Li, Mark T. Bedford, Xiaobing Shi

**Affiliations:** 1 Department of Epigenetics and Molecular Carcinogenesis, The University of Texas MD Anderson Cancer Center, Houston, Texas 77030, USA; 2 Center for Cancer Epigenetics, The University of Texas MD Anderson Cancer Center, Houston, Texas 77030, USA; 3 Department of Molecular and Cellular Biology, Dan L. Duncan Cancer Center, Baylor College of Medicine, Houston, Texas 77030, USA; 4 Department of Pharmacology, University of Colorado School of Medicine, Aurora, Colorado 80045, USA; 5 The University of Texas Graduate School of Biomedical Sciences, Houston, Texas 77030, USA

## Abstract

The euchromatin histone methyltransferase 2 (also known as G9a) methylates histone H3K9 to repress gene expression, but it also acts as a coactivator for some nuclear receptors. The molecular mechanisms underlying this activation remain elusive. Here we show that G9a functions as a coactivator of the endogenous oestrogen receptor α (ERα) in breast cancer cells in a histone methylation-independent manner. G9a dimethylates ERα at K235 both *in vitro* and in cells. Dimethylation of ERαK235 is recognized by the Tudor domain of PHF20, which recruits the MOF histone acetyltransferase (HAT) complex to ERα target gene promoters to deposit histone H4K16 acetylation promoting active transcription. Together, our data suggest the molecular mechanism by which G9a functions as an ERα coactivator. Along with the PHF20/MOF complex, G9a links the crosstalk between ERα methylation and histone acetylation that governs the epigenetic regulation of hormonal gene expression.

## Abstract (web summary)

The histone methyltransferase G9a methylates histone H3K9 to repress gene expression, but it also acts as a coactivator for some nuclear receptors. Here, Zhang *et al*. show that methylation of ERα by G9a recruits the PHF20/MOF complex that deposits histone H4K16 acetylation promoting active transcription.

## Graphical abstract

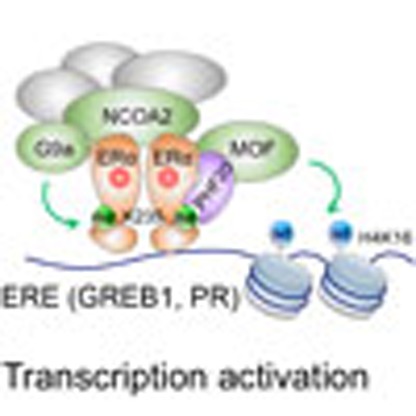

Covalent post-translational modifications (PTMs), such as methylation and acetylation of histones play an essential role in regulating chromatin-associated processes such as transcription^1^. These reversible modifications are catalysed by a number of histone-modifying enzymes. These include histone lysine acetyltransferases (HATs), histone deacetylases, lysine methyltransferases (KMTs) and lysine demethylases, which create a dynamic ‘code' on histones that serves to recruit ‘reader' proteins and their associated chromatin regulators. In the past decades, much effort has been focused on elucidating the functions and mechanisms of these enzymes in modifying histones. However, increasing evidence has demonstrated that these histone-modifying enzymes also act on non-histone proteins, extending their regulatory potential^2^.

Oestrogen receptor α (ERα) is a member of the nuclear hormone receptor family that controls cellular responses to oestrogen^3^. Similar to other ligand-dependent transcription factors, activation of ERα by hormonal signals involves multiple steps, including protein dimerization, nuclear translocation, DNA binding and recruitment of coregulators, which ultimately lead to transcriptional alterations. The nuclear receptor coregulators include both nuclear receptor coactivators (NCOAs) and nuclear receptor corepressors that promote gene activation or repression, respectively, by modulating histone modifications^4^,^5^. For instance, most coactivator complexes contain HATs that deposit acetylation marks on histones to help open up chromatin to increase the accessibility of the underlying DNA to the transcriptional machinery.

In addition to modifying histones, these nuclear receptor coregulators can modify non-histone proteins including ERα. For example, p300/CBP acetylates ERα on several lysine residues in the hinge region: acetylation on ERα K266/288 enhances ERα target gene expression, whereas acetylation at K302/303 inhibits ERα target gene expression^6^,^7^. ERα also undergoes several other PTMs, including phosphorylation, ubiquitylation and sumoylation, which regulate the subcellular localization, protein stability and hormone sensitivity of ERα. These PTMs on ERα protein are associated with distinct biological and clinical outcomes, and thus may serve as prognostic markers for clinical disease. For example, phosphorylation of ERα on serine (S) 305 is associated with tamoxifen resistance, whereas phosphorylation of ERα on S118 and S167 is correlated with better clinical outcomes^8^.

Compared with what is known about the phosphorylation and acetylation of ERα, very little is known about the protein methylation of ERα. In 2008, the first report identifying an ERα methylation event showed that SET7/9 methylates ERα at K302 and modulates ERα protein stability^9^. ERα is also methylated on arginine 260 by the protein arginine methyltransferase 1 to regulate non-genomic functions of ERα in the cytoplasm^10^. We previously screened ∼30 KMTs and found that SMYD2, an H3K4 and H3K36 methyltransferase, specifically methylated ERα at K266 in the hinge region and attenuated the transactivation activity of ERα^11^. In the same screen, we also identified several other enzymes that methylate ERα, including G9a and G9a-like protein (GLP, aka EHMT1).

G9a belongs to the SET domain-containing Su(var)3–9 family of proteins that methylate histone H3K9 (ref. 12). G9a and its closely-related paralogue, GLP, are the primary enzymes that deposit mono- and dimethylation on histone H3K9 in euchromatin, leading to gene silencing^13^. G9a is ubiquitously expressed, and a large body of evidence indicates that G9a is important for diverse cellular processes such as proliferation, differentiation, senescence and replication. Both G9a and GLP are essential for mouse development; knockout of either G9a or GLP leads to mouse embryonic lethality^14^. In addition, the *G9a* and *GLP* genes are upregulated in various types of human cancers, and knockdown of *G9a* suppresses tumour cell growth both *in vitro* and in nude mice^15^. Although many of its biological functions are attributed to its transcriptional corepressor activity, G9a also functions as a coactivator when it is associated with nuclear receptors^16^,^17^,^18^. However, it remains unknown how G9a switches between its roles as a transcriptional corepressor and coactivator.

In the current study, we show that G9a is a coactivator of ERα in breast cancer cells. The coactivator function of G9a is at least partially mediated through the direct methylation of ERα protein at K235. ERαK235 dimethylation (ERαK235me2) is recognized by the Tudor domain of PHF20, which recruits the MOF HAT complex to ERα target gene promoters to acetylate histone H4K16, thus activating a transcriptional program that is essential for the proliferation and survival of ER-positive breast cancer cells. Our study not only identifies a novel methylation event on ERα protein but also uncovers the molecular mechanism by which G9a functions as an ERα coactivator by linking the methylation of a non-histone protein to histone acetylation, thereby activating hormonal gene expression.

## Results

### G9a is an ERα coactivator

**Figure 1:**
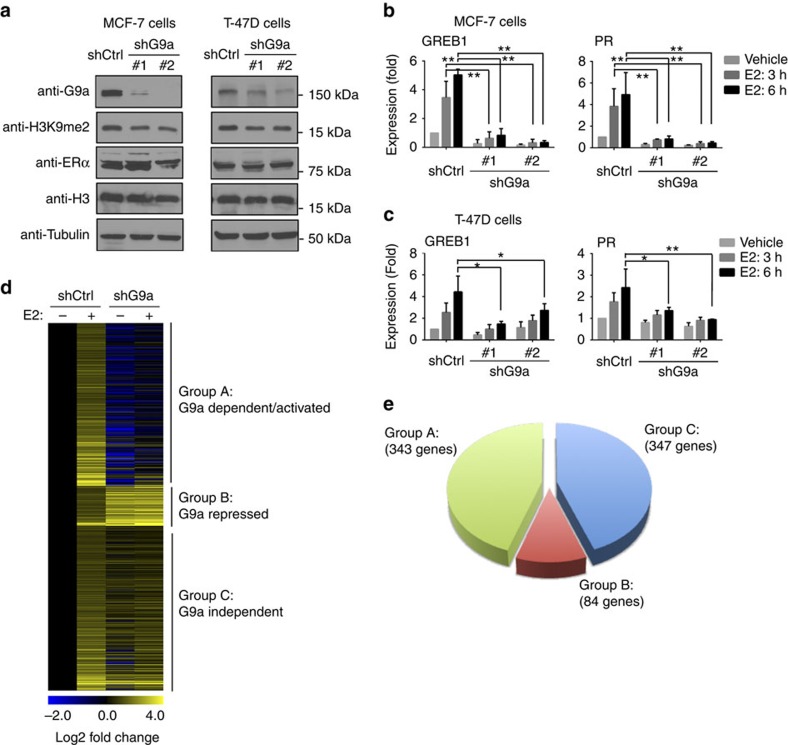
G9a is required for the E2-induced expression of endogenous ERα target genes in breast cancer cells. (**a**) Western blot analysis of G9a protein levels and H3K9me2 levels in control (shCtrl) and G9a knockdown (shG9a) MCF-7 (left) and T-47D (right) cells. Tubulin and total H3 were used as loading controls. (**b**,**c**) G9a is required for E2-induced activation of *GREB1* and *PR*. qPCR analysis of gene expression in control (shCtrl) and G9a knockdown (shG9a) MCF-7 (**b**) and T-47D (**c**) cells treated with 10 nM of E2 for 3 or 6 h. Gene expression was normalized to GAPDH and is shown as fold relative to the expression of each gene in the control cells without E2 treatment that was arbitrarily set as ‘1'. Error bars indicate the mean ± s.e.m. of three experiments. Significant fold changes are indicated as follows: *P<0.05; ^**^P<0.01 (Student's *t-*test). (**d**) Gene expression heatmap of the E2-activated genes in control and G9a knockdown MCF-7 cells ±E2. Heatmap values represent the log2 fold change of read counts relative to the counts in the control cells without E2 induction (lane 1). E2-activated genes are divided into three groups: group A: G9a-dependent genes (downregulated in G9a knockdown cells); group B: G9a-repressed genes (further upregulated in G9a knockdown cells); and group C: G9a-independent genes (no change in G9a knockdown cells) from top to bottom. (**e**) Venn diagram showing the numbers of E2-activated genes assigned to each of the three groups as defined in **d** in MCF-7 cells. *P*<1e-143 (Fisher's exact test).

G9a has been previously reported to be a coactivator of several nuclear receptors that synergistically cooperate with other nuclear receptor coactivators such as NCOA2, coactivator-associated arginine methyltransferase 1 (CARM1) and p300 to activate transcription in a luciferase-based assay^16^. In MCF-7 breast cancer cells, G9a is required for the oestrogen-dependent activation of some ERα target genes, such as *GREB1* and *TFF1* (also known as *pS2*)^18^. To determine whether G9a is a coactivator of endogenous ERα, we knocked down G9a in two ER-positive breast cancer cell lines, MCF-7 and T-47D, using two independent shRNAs (Fig. 1a; Supplementary Fig. 1a), and examined the expression of three well-characterized ERα target genes (*GREB1*, *PR* and *TFF1*). The cells were cultured in oestrogen-deprived medium for 3 days before treatment with 10 nM of 17β-estradiol (E2), and messenger RNAs were collected 3 and 6 h after E2 treatment. Quantitative real-time RT–PCR (qPCR) measurements revealed that compared with the untreated cells, the expression of *GREB1, PR* and *TFF1* showed ∼4- and 6-fold inductions on E2 stimulation for 3 and 6 h in the control MCF-7 cells, respectively. In contrast, G9a-depleted cells showed only a 2- to 3-fold induction of these genes following E2 treatment (Fig. 1b; Supplementary Fig. 1b). Similarly, the E2-induced gene activation was also drastically decreased on G9a depletion in T-47D cells (Fig. 1c; Supplementary Fig. 1b), suggesting that G9a is required for the E2-induced expression of the three ERα target genes we tested.

To discover all genes whose E2-activated expression depends on G9a, we performed RNA-seq analysis in control and G9a knockdown MCF-7 cells with (E2+) or without (E2−) E2 treatment (10 nM, 3 h). Among the 774 E2-activated genes (Supplementary Data 1), 44% (343 genes) were downregulated in the G9a knockdown E2+ cells (Fig. 1d, lane 4) compared with the control E2+ cells (lane 2). Since their E2-induced activation requires G9a, we named these genes as G9a-dependent/activated genes (Fig. 1d,e, Group A). In contrast, ∼11% genes (84 genes) were further upregulated in the G9a knockdown E2+ cells (Group B: G9a repressed genes), whereas 347 genes remained the same E2 response as in the control E2+ cells (Group C: G9a independent genes) (Fig. 1d,e). qPCR analysis of a number of genes randomly selected from the list of G9a-dependent genes demonstrated that G9a is required for their E2-induced expression (Supplementary Fig. 1c). Gene ontology analysis (DAVID) revealed that these genes were enriched in those important for tissue morphogenesis, epithelium development and cell surface receptor-linked signal transductions and so on (Supplementary Table 1; Supplementary Data 2). Taken together, these data suggest that G9a functions as an ERα coactivator for a subset of ERα-regulated genes.

Next, we sought to determine whether the methyltransferase activity of G9a is required for its co-activation of endogenous ERα target genes. We treated MCF-7 cells with a selective G9a inhibitor, BIX-01294 (ref. 19) and assessed ERα target gene expression. The enzymatic activity of endogenous G9a was effectively inhibited by treatment with 4 and 8 μM of BIX-01294 for 24 h, as indicated by western blot analysis of global histone H3K9 methylation levels and the derepression of *MAGE-A2,* a known G9a-repressed gene^20^ (Supplementary Fig. 2a,b). Importantly, under these conditions, although a residual E2 response was sufficient to maintain *GREB1* gene expression, the E2-induced activation of *GREB1* and *PR* were clearly impaired by BIX-01294 in a dose-dependent manner (Supplementary Fig. 2c), suggesting that the catalytic activity of G9a is, at least in part, required for its function as an ERα coactivator.

### G9a methylates ERα protein *in vitro* and in cells

**Figure 2:**
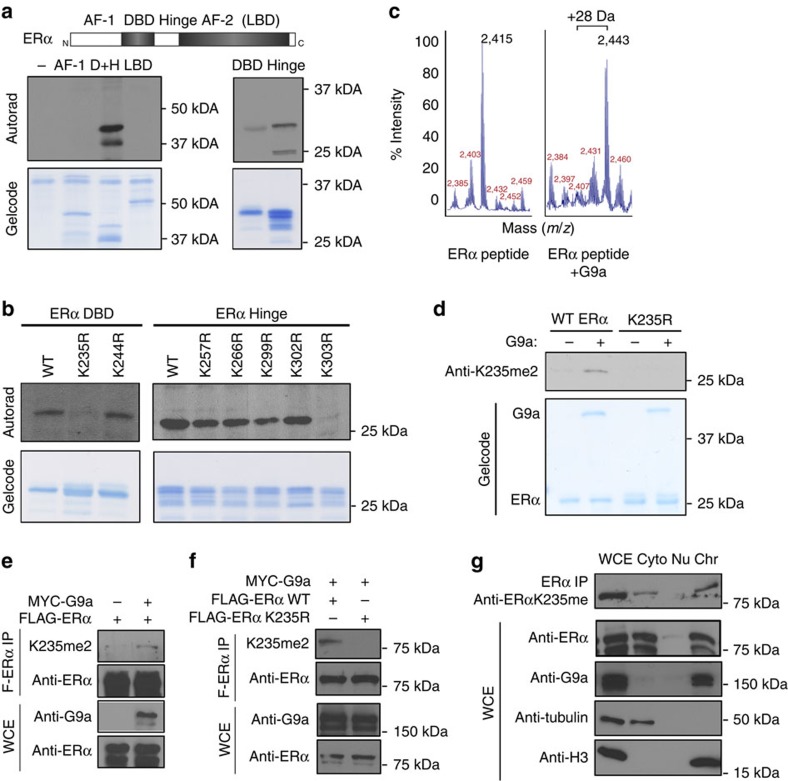
G9a methylates ERα protein *in vitro* and in cells. (**a**,**b**) G9a methylates ERα at K235 and K303 *in vitro*. *In vitro* methylation assays were performed as described in the Methods section and assessed by autoradiograph. The top panel of **a** shows the diagram of the ERα protein domains. AF-1: activation function 1 region; DBD: DNA-binding domain; D+H: DBD+hinge; H: hinge region; LBD: ligand-binding domain.Recombinant ERα protein fragments (**a**) or fragments containing the indicated ERα point mutants (**b**) were incubated with recombinant G9a in the presence of ^3^H-labelled *S*-adenosylmethione. GelCode blue staining was used to assess input protein levels. (**c**) G9a dimethylates ERα at K235. Mass spectrometric analysis of the ERα peptide (aa 227–244) with or without G9a incubation. The peptide masses are shown; a change in mass of 28 Da indicates the addition of two methyl groups. (**d**) The anti-ERαK235me2 antibody specifically recognizes ERα protein methylated by G9a at K235 *in vitro*. The anti-ERα-K235me2 antibody was assessed by western blotting using recombinant ERα protein methylated by G9a. The K235R mutant was used as a negative control. GelCode blue staining shows the relative amounts of proteins used in the methylation assay. (**e**,**f**) The anti-ERαK235me2 antibody recognizes G9a-methylated ERα protein at K235 in cells. Western blot analysis of Flag-IPed ERα from HEK-239T cells co-transfected with Flag-ERα and Myc-G9a or vector control (**e**) or with the Myc-G9a and either WT ERα or the ERαK235R mutant (**f**). ERα protein levels in whole cell extract (WCE) and IP samples were used as loading controls. (**g**) Methylated ERα is enriched in the chromatin fraction. Top panel, western blot analysis of Flag-ERα immunoprecipitated from the cytoplasmic (Cyto), soluble nuclear (Nu) and chromatin-associated (Chr) fractions of cells probed with the anti-ERαK235me2 antibody. HEK-239T cells were co-transfected with Flag-ERα and Myc-G9a and were cultured in regular DMEM medium containing phenol red. Cell fractionation was assessed using anti-tubulin (cytosolic marker) and anti-histone H3 (chromatin marker) antibodies. The distribution of ERα and G9a in the different cellular fractions is shown.

G9a has been shown to directly methylate several transcription factors to regulate their activity^21^,^22^,^23^,^24^,^25^. Because G9a functions as an ERα coactivator in a methyltransferase activity-dependent manner, and because ERα protein is subject to extensive PTMs, including methylation^8^. We hypothesized that G9a regulates ERα transactivation activity by directly methylating the ERα protein. To test this hypothesis, we cloned and purified three ERα fragments: (1) the N-terminal activation function domain (AF-1); (2) the DNA-binding domain (DBD) and linked hinge region; and (3) the C-terminal ligand-binding domain (LBD) of ERα (Supplementary Fig. 3a), and incubated them with recombinant G9a SET domain and ^3^H-labelled methyl donor *S*-adenosylmethionine in an *in vitro* methylation assay. We found that G9a specifically methylated the DBD and hinge fragment, but not the AF-1 and LBD fragments. Methylation assays using smaller protein fragments revealed that G9a methylated both the DBD and the hinge region (Fig. 2a).

To identify the specific ERα lysine residues that are methylated by G9a, we mutated several DBD and hinge region lysine (K) residues to arginine (R). We focused on lysine residues within RK/ARK sequences, previously reported to be a G9a recognition motif^24^. These mutants were then used as substrates in G9a methylation assays. Two mutants, K235R and K303R, abolished G9a methylation of the ERα DBD and the hinge regions, respectively, suggesting that G9a can methylate these two residues *in vitro* (Fig. 2b). Mass spectrometric analysis of two G9a methylated peptides, spanning either ERα amino acids 227–244 or 294–313, revealed an increase in mass of 28 Da to the ERα peptide containing K235 and an increase of 14 Da to the peptide containing K303 (Fig. 2c; Supplementary Fig. 3b), suggesting that G9a dimethylates ERα at K235 and monomethylates ERαK303 *in vitro*.

Sequence alignments revealed that the consensus G9a recognition motif around ERα K235 and K303 are partially conserved between ERα and several other nuclear receptors in humans (Supplementary Fig. 3c). To determine whether either of these residues is important for G9a-dependent ERα transactivation activity, we mutated each residue to R and tested these G9a methylation-deficient ERα mutants in an oestrogen response element (ERE)-luciferase reporter assay. We found that ERαK235R was impaired in transactivation activity (Supplementary Fig. 3d); in contrast, the ERαK303R mutant exhibited an increase in transactivation relative to the wild-type (WT) ERα control, which is consistent with previous reports^26^,^27^. To further demonstrate the importance of ERαK235 methylation in transcriptional activation, we repeated the ERE-luciferase assays using WT G9a, a G9a H1113K mutant that lacks methyltransferase activity^28^, and WT ERα and the K235R mutant. The results revealed that although G9a had both a catalytic activity-dependent and -independent role in promoting ERα transactivation activity, the moderate catalytic activity-dependent coactivator function of G9a was largely dependent on NCOA2 and ERαK235 (Supplementary Fig. 3e,f).

Next, we sought to determine whether G9a could methylate ERα in cells. We generated a polyclonal anti-ERαK235me2 antibody that specifically recognized ERαK235me2, but not other G9a methylated proteins (for example, H3K9me2 and p53K373me2), as shown by peptide dot blot analysis (Supplementary Fig. 3g). The recognition of ERα by the anti-K235me2 antibody was methylation dependent, as it recognized recombinant WT ERα protein incubated with G9a but not unmethylated ERα or the ERα K235R mutant (Fig. 2d). However, we were unable to detect endogenous ERαK235me2 in MCF-7 and T-47D cells by western blot analysis, perhaps due to the low affinity of the antibody for the substrate or the low abundance of ERαK235me2 in these cells. To enrich for methylated ERα proteins in cells, we immunoprecipitated (IP) Flag-tagged ERα overexpressed in HEK 293T cells with or without G9a coexpression. Western blot analysis of the IPed Flag-ERα probed with the anti-ERαK235me2 antibody showed an increased methylation signal in the cells co-transfected with G9a (Fig. 2e). Importantly, we did not detect any signal on the IPed Flag-ERαK235R mutant, demonstrating that the increased signal was ERαK235 methylation specific (Fig. 2f). Furthermore, cell fractionation assays and immunofluorescence experiments revealed that the methylated ERα was largely in the nucleus and associated with chromatin (Fig. 2g; Supplementary Fig. 3h). Taken together, these results suggest that G9a methylates ERα at K235 in the nucleus.

### G9a regulates the growth of ERα-positive breast cancer cells

The rapid and effective response of ERα to hormonal signalling is critical to the activation of a transcription program that is essential for the proliferation of ER-positive breast cancer cells. Because G9a depletion greatly reduced the expression of a large subset of E2-induced genes (Fig. 1d,e), we hypothesized that G9a plays an essential role in regulating the growth properties of breast cancer cells. To test this, we first assessed the proliferation of ER-positive breast cancer cells on G9a depletion. We found that the depletion of G9a by two independent shRNAs greatly reduced cell growth of both MCF-7 and T-47D cells (Supplementary Fig. 4a,b). Next, we conducted colony formation assays using MCF-7 cells and observed a drastic reduction in colony numbers of the G9a knockdown cells compared with the control cells (Supplementary Fig. 4c,d). To assess the impact of G9a depletion on anchorage-independent cell growth, we performed soft agar colony formation assays. We found that G9a-depleted cells formed substantially fewer colonies than control cells (Supplementary Fig. 4e). Taken together, these results suggest that G9a is required for maintaining the growth properties of these two ER-positive breast cancer cells.

Next, we determined whether inhibiting the methyltransferase activity of G9a could effectively suppress cancer cell growth and survival. We treated MCF-7 cells with different doses of the selective G9a inhibitor BIX-01294 and counted cell numbers for 6 days. We found that BIX-01294 inhibited cell proliferation in a dose-dependent manner (Supplementary Fig. 4f). In addition, treating MCF-7 cells with 0.5 or 1 μM of BIX-01294 greatly suppressed colony formation (Supplementary Fig. 4g,h). Importantly, another G9a-specific inhibitor has also been found to inhibit the growth of breast cancer cells^29^, suggesting that G9a-selective inhibitors have therapeutic potential for ER-positive breast cancers.

### PHF20 Tudor domain recognizes ERαK235me2

**Figure 3:**
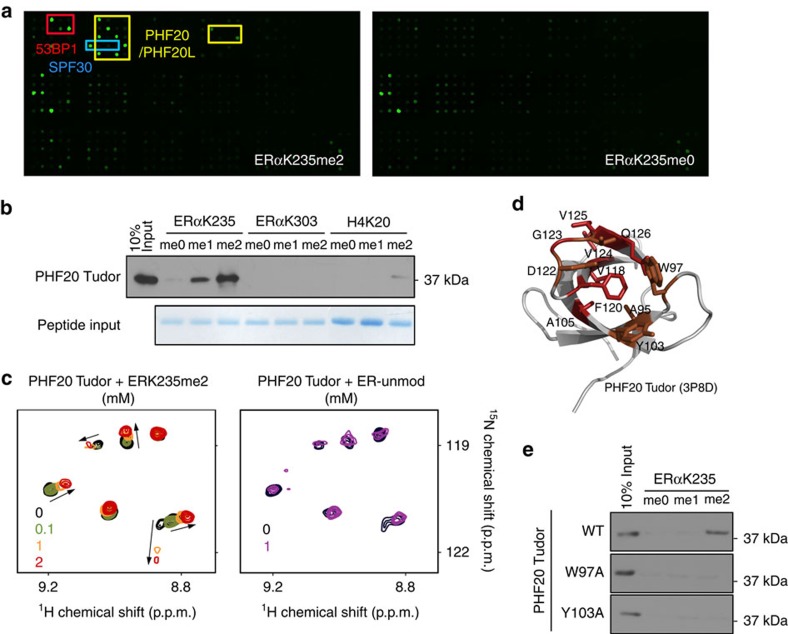
ERαK235me2 is recognized by the PHF20 Tudor domain. (**a**) Identification of ERαK235me2-specific readers by the chromatin-associated domain array (CADOR). The array probed with unmodified ERαK235 peptide was used as a negative control. Positive interactions of the ERαK235me2 peptide are highlighted in coloured boxes. Red: 53BP1 Tudor; Blue: *S. pombe* SPF30 Tudor; Yellow: PHF20/20L Tudors. The list of all the domains on the CADOR is shown in Supplementary Fig. 5. (**b**) The PHF20 Tudor domain binds to ERαK235me peptides *in vitro*. Western blot analysis of peptide pulldowns of PHF20 Tudor using the indicated ERα and histone peptides. GelCode staining shows the peptide input for the assay. (**c**) Overlays of ^1^H,^15^N HSQC spectra of PHF20 Tudor collected as the ERαK235me2 peptide (left) or unmodified ER peptide (right) were titrated in. The spectra are colour coded according to the peptide concentration (inset). (**d**) A ribbon diagram of the structure of PHF20 Tudor (ID: 3P8D). The residues that exhibit large ERαK235me2-induced resonance changes (red) or resonance broadening (brown) are highlighted. (**e**) PHF20 Tudor binding to the ERαK235me2 peptide is methylation dependent. Western blot analysis of ERαK235me peptide pulldowns of the WT PHF20 Tudor domain or two methyl-binding-deficient mutants (W97A and Y103A).

Methylation on histones provides docking sites for ‘reader' proteins, which recruit additional epigenetic regulators to modulate chromatin dynamics. Increasing evidence suggests that methylation on non-histone proteins also functions through a similar mechanism. Thus, we hypothesized that ERαK235me2 modulates protein–protein interactions between ERα and readers that can recognize this mark. To identify readers of ERαK235me2, we used an ERα peptide (aa 227–244) bearing dimethylated K235 to probe a chromatin-associated domain array that contains over 300 known and potential epigenetic reader domains (Supplementary Fig. 5)^30^. We identified several Tudor domains that recognized the ERα peptide in a K235 methylation-dependent manner, including the Tudor domains of 53BP1, *S. pombe* SPF30 and PHF20/PHF20L (Fig. 3a).

Because the second Tudor domain of PHF20/PHF20L has been shown to recognize dimethylation on several histones and non-histone proteins that regulate diverse nuclear processes^31^,^32^,^33^,^34^, we sought to determine whether this Tudor domain is capable of interacting with ERαK235me2. First, we performed peptide pull-down assays using the purified PHF20 Tudor and biotin-labelled methylated ERα or histone peptides. We detected a strong interaction between PHF20 Tudor and the ERαK235me2 peptide but not with the methylated ERαK303 and H4K20 peptides (Fig. 3b). To characterize the interaction between PHF20 Tudor and ERαK235me2 peptide in more detail, we performed ^1^H,^15^N Heteronuclear Single Quantum Coherence (HSQC) NMR experiments. The ^15^N-labelled PHF20 Tudor exhibited large chemical shift changes when the ERαK235me2 peptide was gradually added, whereas no resonance perturbations were observed on addition of the unmethylated ERα (aa 228–239) peptide (Fig. 3c), suggesting that PHF20 Tudor interacts only with the dimethylated ERα species.

Interaction with ERαK235me2 led to significant shifting and broadening of backbone amide resonances of the A95, W97, Y103, A105, V118, F120, D122-Q126 residues of PHF20 Tudor. Mapping the most perturbed residues on the crystal structure of PHF20 Tudor (PDB: 3P8D)^31^ revealed that these residues are clustered at one of the open ends of the domain's β-barrel (Fig. 3d). The W97, Y103 and F120 residues at the top of the β-barrel likely form an aromatic cage around the dimethylammonium group of K235, in a manner similar to how this cage holds dimethylated lysine residues of p53 peptides^31^. An overall comparable pattern of chemical shift perturbations caused by ERαK235me2 and dimethylated p53 peptides^31^ suggests strong conservation of the mechanism for dimethyllysine recognition by the PHF20 Tudor domain. As expected, mutations of the two aromatic residues W97 and Y103 abolished the binding of PHF20 Tudor to ERαK235me2 in peptide pull-down assays (Fig. 3e), thus reinforcing our finding that PHF20 Tudor–ERα interaction is methylation dependent.

**Figure 4:**
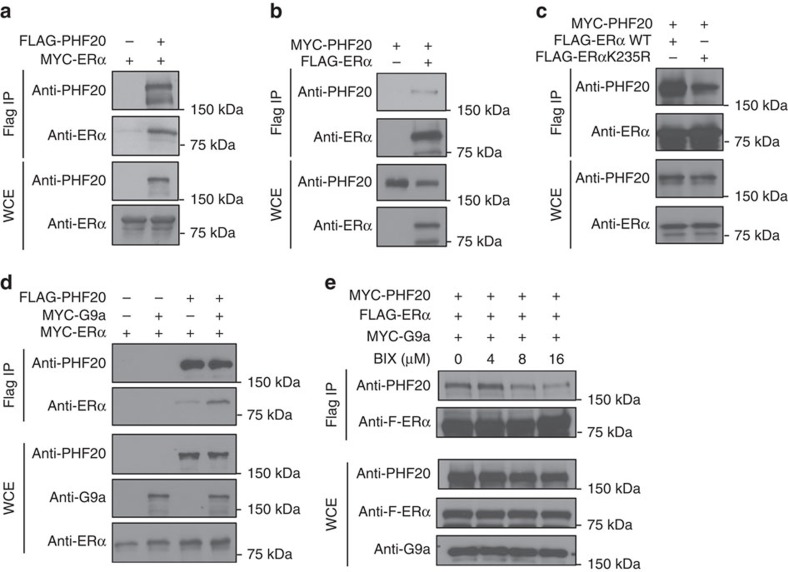
Methylation-dependent interaction between ERα and PHF20 in cells. (**a**,**b**) PHF20 interacts with ERα in cells. Western blot analysis of reciprocal co-IP experiments in HEK-293T cells co-transfected with Flag-PHF20 and Myc-ERα (**a**) or Flag-ERα and Myc-PHF20 (**b**). WCE and immunoprecipitated Flag-tagged proteins were probed with the indicated antibodies. (**c**) ERαK235R mutation reduces the interaction between ERα and PHF20 in cells. Western blot analysis of co-IP experiments in HEK-293T cells co-transfected with Myc-PHF20 and either Flag-ERα or the Flag-ERαK235R mutant. (**d**) G9a enhances the interaction between ERα and PHF20 in cells. Western blot analysis of co-IP experiments using HEK-293T cells co-transfected with Flag-PHF20 and Myc-ERα ±G9a. (**e**) The G9a inhibitor BIX-01294 (BIX) diminishes the interaction between ERα and PHF20 in cells. Western blot analysis of co-IP experiments using HEK-293T cells co-transfected with Flag-ERα and Myc-PHF20. Cells were treated with the indicated amounts of BIX for 24 h before being collected for co-IP experiments. Western blotting analysis of the WCE shows that the total levels of G9a, PHF20 and ERα were not affected by BIX treatment.

To determine whether PHF20 associates with ERα in cells, we performed reciprocal co-IP experiments and found that PHF20 robustly binds to ERα protein *in vivo* (Fig. 4a,b). Mutation of K235 to R drastically reduced the PHF20–ERα interaction (Fig. 4c), demonstrating the importance of this residue. Overexpression of G9a, which increased ERαK235me2 levels in cells (Fig. 2e), enhanced the interaction between PHF20 and ERα (Fig. 4d), whereas inhibition of G9a catalytic activity by BIX-01294 treatment markedly diminished the PHF20–ERα interaction (Fig. 4e). It is worth noting that notable interaction was detected between PHF20 and the ERα K235R mutant (Fig. 4c), suggesting that in addition to G9a-mediated ERα K235 methylation, other mechanisms contribute to their protein–protein interactions.

### PHF20 regulates activation of a subset of E2-induced genes

**Figure 5:**
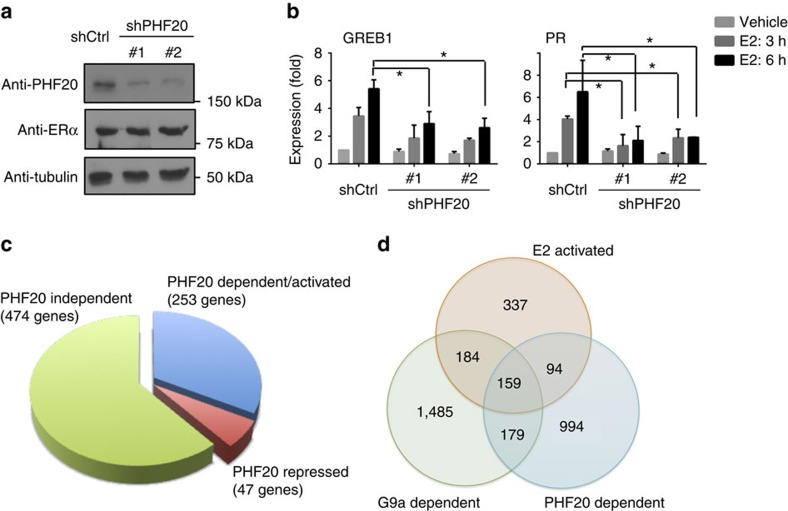
PHF20 is required for the E2-induced expression of endogenous ERα target genes. (**a**) Western blot analysis of PHF20 levels in control (shCtrl) and PHF20 knockdown (shPHF20) cells. Tubulin was used as a loading control. (**b**) PHF20 is required for E2-incuded expression of *GREB1* and *PR*. qPCR analysis of gene expression in control and PHF20 knockdown cells treated with 10 nM of E2 for 3 or 6 h. Error bars indicate the mean ±s.e.m. of three experiments. *P<0.05 (Student's *t-*test). (**c**) Venn diagram of the E2-activated genes in MCF-7 cells that are differentially affected by PHF20 knockdown. Approximately 33% of E2-induced genes require PHF20 for gene activation. *P*<1e-104 (Fisher's exact test). (**d**) Venn diagram showing the E2-activated genes in MCF-7 cells that are differentially affected by G9a knockdown or PHF20 knockdown. About 20% of E2-induced genes require both G9a and PHF20 for gene activation. The *P* value of the overlap between G9a-dependent and PHF20-dependent E2-activated genes is <1e-300 (Fisher's exact test). The genes of each group are listed in Supplementary Data 1.

To determine whether PHF20 is required for ERα target gene expression, we carried out ERE-luciferase assays by co-expressing PHF20 with the WT ERα or ERαK235R mutant. We found that PHF20 can function as an ERα coactivator and this function largely depended on ERαK235 (Supplementary Fig. 6a). Next, to determine whether PHF20 is required for the activation of endogenous ERα target genes, we knocked down PHF20 in MCF-7 cells using two independent shRNAs (Fig. 5a; Supplementary Fig. 6b), and assessed the expression of ERα target genes by qRT–PCR. Indeed, the E2-induced activation of *GREB1* and *PR* was drastically decreased, whereas *ESR1* gene expression was not affected on PHF20 depletion (Fig. 5b; Supplementary Fig. 6c).

To determine the ERα coactivator function of PHF20 genome wide, we further performed RNA-seq analysis in control and PHF20 knockdown MCF-7 cells ±E2 treatment. Among the 774 E2-induced genes, >30% (253 genes) were downregulated in the PHF20 knockdown cells compared with the control cells, and we named them as PHF20-dependent/activated genes (Fig. 5c; Supplementary Data 2). In contrast, ∼6% genes (47 genes) were further upregulated on PHF20 depletion (PHF20-repressed genes), whereas 474 genes remained the same E2 response as in the control E2+ cells (PHF20-independent genes) (Fig. 5c). Gene ontology analysis revealed that the PHF20-dependent E2-activated genes were enriched in cell adhesion, intracellular signalling, and epithelium development (Supplementary Table 2; Supplementary Data 2). Comparison of the G9a-dependent, PHF20-dependent and E2-activated genes showed that these three groups influenced the expression of a shared group of 159 genes (Fig. 5d). These overlapping genes were enriched in the GO categories of epithelium development, and tube morphogenesis and development (Supplementary Table 3). qRT-PCR analysis demonstrated that PHF20 is required for the E2-induced expression of the overlapped genes, but not for genes from the other groups (Supplementary Fig. 6d,e).

### PHF20/MOF is required for deposition of H4K16 acetylation

PHF20 and PHF20L are components of the MOF histone acetyltransferase complex^35^, which acetylates histone H4K16 and certain non-histone proteins that regulate transcription and ATM-dependent DNA damage response in human cells and dosage compensation in *Drosophila*^36^. Mice null for PHF20 generally die before weaning and have a deregulated MOF signalling pathway^34^. The PHF20/MOF complex physically interacts with the MLL histone H3K4 KMT complex during active transcription, and the activities of both complexes are required for optimal transcription activation^37^. Interestingly, the MLL complex has been shown to function as an ERα coactivator^38^,^39^. Because PHF20 recognizes ERαK235me2 and associates with the MOF complex, we hypothesized that the recognition of G9a-mediated ERαK235me2 by PHF20 facilitates the recruitment of the MOF complex to ERα target genes to deposit histone H4K16 acetylation (H4K16ac).

**Figure 6:**
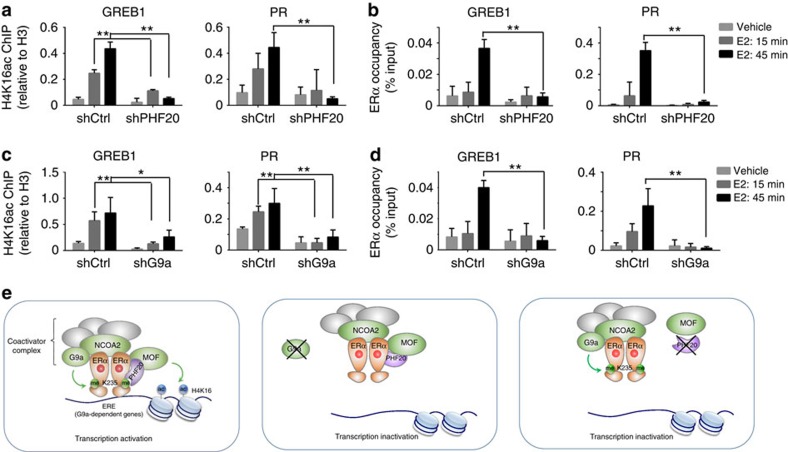
PHF20 is required for the deposition of histone H4K16ac on ERα target genes. (**a**) PHF20 is required for the E2-induced accumulation of histone H4K16ac on the promoters of *GREB1* and *PR*. qPCR analysis of histone H4K16ac ChIP in control and PHF20 knockdown cells 15 and 45 min after E2 treatment. The H4K16ac ChIP was calculated as a ratio relative to total H3 ChIP. (**b**) PHF20 is required for ERα chromatin recruitment. qPCR analysis of ERα ChIP in the cells as in **a**. ERα ChIP is shown as a ratio relative to input. (**c**) G9a is required for the E2-induced accumulation of histone H4K16ac on the promoters of *GREB1* and *PR*. qPCR analysis of histone H4K16ac ChIP in control and G9a knockdown cells 15 and 45 min after E2 treatment. (**d**) G9a is required for ERα chromatin recruitment. qPCR analysis of ERα ChIP in the cells as in **c**. In **a**–**d**, all error bars indicate the mean ±s.e.m. of three experiments. *P<0.05, ^**^P<0.01 (Student's *t-*test). (**e**) Working model of G9a-mediated ERα methylation in hormonal response. Left panel: G9a methylates ERα at K235 in the nucleus in response to E2 stimulation, and ERαK235me2 is recognized by the Tudor domain of PHF20, which recruits the MOF complex to acetylate histone H4K16, thereby promoting the expression of ERα target genes *GREB1* and *PR*. Middle panel: depletion of G9a abolishes ERαK235 methylation and the methylation-dependent ERα–PHF20 interaction, thereby reducing the recruitment of PHF20/MOF and ERα to the EREs of ERα target genes *GREB1* and *PR*, consequently reducing their expression. Right panel: depletion of PHF20 reduces the H4K16ac levels on chromatin and the recruitment of ERα to EREs via feedback regulation, thereby reducing the expression of ERα target genes *GREB1* and *PR.*

To test this hypothesis, we asked whether the reduced gene expression in PHF20 knockdown cells was due to a reduction in MOF occupancy. Since all commercial antibodies against MOF and PHF20 we tested did not work under chromatin IP (ChIP) conditions, we answered this question indirectly by assessing the levels of H4K16ac on the promoters and/or enhancers of ERα target genes. H4K16 is the main target of the MOF complex on histones^36^, therefore changes in H4K16ac levels likely reflect the dynamics of MOF occupancy on chromatin. We performed ChIP experiments to determine the distribution of histone H4K16ac on the ERα target gene promoters in control and PHF20 knockdown MCF-7 cells. We found that E2 treatment induced a dramatic increase in H4K16ac levels on the promoters of *GREB1* and *PR*, whereas no apparent H4K16ac signals were detected on genes that are not regulated by PHF20 (Fig. 6a and Supplementary Fig. 6f). Importantly, on PHF20 depletion, such an E2-dependent increase of H4K16ac on *GREB1* and *PR* promoters was greatly diminished (Fig. 6a), suggesting that PHF20 is essential for the deposition of histone H4K16ac on PHF20-activated ERα target genes.

Disruption of the recruitment of a coactivator can lead to perturbations in the docking of ERα on target genes^40^. Therefore, we tested whether decreased H4K16ac levels in PHF20 knockdown cells affects ERα occupancy on target genes. ERα ChIP experiments revealed an E2-dependent increase of ERα on the promoters of *GREB1* and *PR*; however, no or minimal ERα occupancy was detected in the PHF20 knockdown cells (Fig. 6b), suggesting that PHF20 or PHF20/MOF-dependent H4K16ac is important for the recruitment of ERα to chromatin.

Finally, we asked whether the PHF20-mediated H4K16 acetylation and the recruitment of ERα to chromatin depend on G9a. To address this question, we performed ChIP to determine H4K16ac levels and ERα occupancy on target genes in G9a-depleted cells. Similar to what we observed in PHF20 knockdown cells, G9a depletion markedly reduced the levels of both H4K16ac and ERα on the promoters of *GREB1* and *PR* (Fig. 6c,d). Taken together, these results suggest that G9a is required for E2-depenedent activation of ERα target genes and PHF20-mediated H4K16 acetylation, and this regulation is, in part, through G9a-mediated methylation of ERα protein (Fig. 6e).

## Discussion

G9a has been well characterized as a transcriptional corepressor for a large number of transcription factors through its methylation of histone H3K9 in euchromatin^41^. However, the Stallcup group has reported an unconventional function of G9a as a coactivator of several nuclear receptors, including the glucocorticoid receptor, androgen receptor and ERα (refs 16, 17, 18, 42). They showed that G9a synergistically cooperates with other coactivators including CARM1 and p300, and that the enzymatic activity of G9a is largely not required in a transient transfection assay. In the current study, we used different approaches to assess the expression of endogenous ERα target genes in MCF-7 cells and found that the coactivator function of G9a at least partially depends on its catalytic activity. Treating cells with the G9a-specific inhibitor BIX-01294 greatly reduced E2-dependent gene activation. However, there was still an E2-dependent response in the BIX-01294-treated cells, although the induction was lower than that of the untreated cells. These data suggest that similar to the dual role of the histone H3K27 methyltransferase EZH2 in regulating gene expression in castration-resistant prostate cancer^43^, G9a has both methyltransferase activity-dependent and methyltransferase-independent roles in promoting hormonal gene activation.

The requirement of the methyltransferase activity for the coactivator function of G9a has also been observed in some other cases. For instance, under hypoxic conditions, G9a and GLP specifically methylate pontin, and this methylation increases the recruitment of p300 to potentiate HIF-1α-mediated gene activation^22^. In the current study, we found that G9a methylates ERα at K235, which recruits the PHF20/MOF complex to deposit histone acetylation, thus promoting gene activation. Interestingly, the consensus G9a-recognition sequence is conserved among many nuclear receptors, indicating that G9a may regulate nuclear receptors other than ERα via a similar mechanism. In support of this model, G9a has been reported to methylate a large number of non-histone proteins^24^,^25^,^44^,^45^. Nevertheless, the biological functions and molecular mechanisms of these methylation events await future studies.

The methylation of specific residues on histones facilitates or hinders modifications on other residues. For example, H3K9 methylation is a prerequisite for methylation on H4K20, but it inhibits methylation on H3K4 on the same histone tail^46^. It is conceivable that methylation on non-histone proteins provide a mechanism for crosstalk with other PTMs in a similar way. ERα is subjected to extensive PTMs, including methylation on two lysine residues. Methylation of ERαK302 by SET7/9 inhibits ERα ubiquitylation, thus stabilizing the ERα protein^9^. SMYD2-mediated ERαK266 methylation antagonizes acetylation at the same residue to attenuate ERα transactivation activity^11^. In the vicinity of G9a-methylated ERαK235, S236 can be phosphorylated by protein kinase A. This phosphorylation event is believed to inhibit ERα dimerization and thus prevent ERα from binding to DNA^47^. Since G9a-mediated methylation of ERαK235 is critical to ERα activation, it is likely that K235 methylation and S236 phosphorylation, analogous to histone H3K9 methylation and H3S10 phosphorylation^48^, counteract each other in promoting and attenuating ERα transactivation, respectively.

Our *in vitro* methylation assay revealed that a second site on ERα, K303 in the hinge region, can also be methylated by G9a *in vitro*, but due to the lack of an antibody specific for this modification, its existence has not been confirmed *in vivo*. Nevertheless, a somatic mutation (A908G), which causes a K303R amino-acid change, has been identified in premalignant breast lesions^49^, and in invasive breast cancers among some, but not all races^50^,^51^,^52^,^53^. K303R confers mitogenic hypersensitivity to estrogen and resistance to aromatase inhibitors in cultured cells and accelerates mammary maturation and differentiation in mice^49^,^54^,^55^. K303 and nearby residues in the hinge region are heavily post-translationally modified. These modifications include acetylation and sumoylation on K299/K302/K303, ubiquitylation on K302/K303, methylation on K302 and phosphorylation on S305 (refs 7, 9, 56, 57, 58). Therefore, the cellular and biological consequences of a K303R mutation likely result from the disruption of the crosstalk among these PTMs^8^.

Methylation on histones is known to create docking sites for reader proteins, which in turn recruit additional epigenetic regulators to modulate chromatin dynamics^59^. In the current study, we found that ERαK236me2 recruits the PHF20/MOF complex, driving the crosstalk between ERα protein methylation and histone acetylation. We propose that such a ‘reading and recruiting' model is a general mechanism for methylation on non-histone proteins that is likely mediated by the readers that also recognize histone methylation. This notion is strongly supported by increasing evidence. For example, 53BP1, which was initially identified as an H4K20me2 reader^33^,^60^, recognizes p53K382 and p53K370 methylation^61^,^62^,^63^, and PHF20, originally identified as an H4K20me2 and H3K9me2 reader^33^, also binds to dual methylation on p53K370/K382 (ref. 31). Furthermore, in addition to recognizing methylated p53 and pRb proteins^64^,^65^, the pan-mono- or dimethyllysine-binding protein L3MBTL1 was found to bind over 300 methylated proteins in a proteome-wide study^66^. Together, these data suggest that histone methyllysine readers have a general role in recognizing a broad spectrum of methylated lysines on non-histone proteins. Notably, many reader proteins reside in large protein complexes composed of histone-modifying enzymes or remodelers; therefore it is likely that the reading events ultimately lead to alterations in chromatin-related processes.

In summary, our study uncovers the molecular mechanism by which G9a functions as an ERα coactivator in the oestrogen response pathway. Depletion of G9a protein abolishes the transcriptional response to oestrogen and inhibits cell proliferation and transformation, suggesting that G9a is a potential therapeutic target for the treatment of ER-positive human breast cancers. In this regard, small molecules that selectively inhibit G9a/GLP methyltransferase activity exhibit strong activity against the clonal and metastatic properties of different types of cancer cells^19^,^29^,^67^,^68^. Thus, *in vivo* characterization of these G9a-specific inhibitors and clinical trials of these agents as adjuvant therapy for patients with ER-positive breast cancer are warranted.

## Methods

### Materials

Complementary DNA encoding full-length human ERα, G9a, PHF20 and NCOA2 genes were cloned into pENTR3C and subsequently cloned into destination vectors using the Gateway technique (Invitrogen). The destination vectors used in this study include p3Flag and pCAG-Flag (for Flag-tag expression), pCAG-Myc (for Myc-tag expression) and pCAG (for no-tag expression). pERE-Luc Firefly luciferase and pRl-TK Renilla luciferase were used for the reporter assays. For *in vitro* enzymatic assays, G9a, ERα, ERα fragments and mutants were cloned into pGEX-6P1 (GE Healthcare) to express and purify the proteins as GST-tagged proteins. Point mutations were generated using site-directed mutagenesis (Stratagene). shRNA constructs were purchased from Sigma. The G9a-targeting shRNA sequences used in this study were 5′-CTCCAGGAATTTAACAAGAT-3′, 5′-CTCTTCGACTTAGACAACAA-3′ and 5′-GAGAGAGTTCATGGCTCTTT-3′ and the PHF20-targeting shRNA sequences were 5′-CCGAGAAATACACCTGTTAT-3′ and 5′-CTGATAAAGAAGGAAAGTTA-3′. Peptides were synthesized at the W.M. Keck Facility at Yale University or at CPC-Scientific. Polyclonal anti-dimethylated ERαK235 rabbit sera (CPC Scientific, 1:500) were affinity-purified with an ERαK235me2 peptide and depleted against the corresponding unmethylated peptide. Anti-G9a antibodies were purchased from MBL (D141-3, 1:1,000) and Sigma (G6919, 1:2,000); anti-PHF20 (3934S, 1:1,000) antibody from Cell Signaling; anti-ERα (Sc-503, 1:1,000) and anti-GST (Sc-459, 1:1,000) antibodies from Santa Cruz; anti-FLAG (F-1804, 1:5,000) and anti-tubulin (T8328, 1:5,000) antibodies from Sigma; anti-H4K16ac (07–329, 1:1,000) antibody from Millipore; anti-total H3 (ab1791, 1:5,000) and anti-H3K9me2 (ab1220, 1:1,000) antibodies from Abcam; and streptavidin horseradish peroxidase from Thermo Scientific. 17β-estradiol and the G9a inhibitor BIX-01294 were purchased from Sigma.

### Protein expression and purification

The pGEX constructs encoding human G9a, ERα and ERα fragments including AF1 (aa 1–190), DBD (aa 175–250), hinge (aa 251–310), DBD+hinge (aa 175–310), LBD (aa 305–596) and the second Tudor domain of PHF20 (aa 58–148) were expressed in *E. coli* Rosetta (DE3) pLysS cells. After induction with IPTG (0.5 mM) for 16–18 h at 18 °C, cells were collected by centrifugation at 5,000*g*, lysed by sonication and purified using Glutathione Sepharose 4B beads (GE Healthcare). For the NMR titration assays, PHF20 Tudor was expressed in the ^15^NH_4_Cl-supplemented (Sigma) minimal media, and the GST tag was cleaved with PreScission protease, and the cleaved protein was concentrated using Millipore concentrators (Millipore).

### *In vitro* methylation assays

Recombinant G9a (2 μg) was incubated with recombinant ERα fragments proteins (2 μg) in methylation assay buffer (50 mM Tris-HCl, pH 8.0, 10% glycerol, 20 mM KCl, 5 mM MgCl_2_, 1 mM DTT, 1 mM PMSF and 0.1 mM unlabelled or ^3^H-labelled *S*-adenosylmethionine (GE Healthcare)) at 30 °C for 4 h. Reactions were stopped by adding SDS–PAGE sample buffer, and the methylation status was determined by autoradiography or western blotting analysis. For mass spectrometry analysis, ERα peptides (1 μg) were used as substrates in the methylation assays and mass spectrometry analysis was performed at the Mass Spectrometry Core at the University of Texas Medical Branch at Galveston.

### Peptide pull-down assays and domain array

For peptide pulldown assays, 1 μg of biotinylated histone and ERα peptides with or without methylation were incubated with 1 μg GST-PHF20 Tudor in binding buffer (50 mM Tris-HCl, pH 7.5, 300 mM NaCl, 0.1% NP-40 and 1 mM PMSF) for overnight. Streptavidin beads (GE Healthcare) were added into the mixture and incubated for 1 h with rotation. The beads were then washed three times, and analysed by SDS−PAGE and western blotting. For the protein domain array, the CADOR 5.0 protein domain array was probed with biotinylated ER peptides as described previously^33^. The protein domains spotted on the CADOR 5.0 array are listed in the Supplementary Fig. 5.

### NMR titrations with ER peptides

The ^1^H,^15^N HSQC spectra of 0.2 mM uniformly ^15^N-labelled PHF20 Tudor (in 25 mM Tris-HCl, pH 7.5 buffer, 150 mM NaCl, 5 mM dithiothreitol and ∼8% D_2_O) were collected at 298 K on a Varian INOVA 600 MHz spectrometer. Binding was monitored by titrating unmodified ERα and ERαK235me2 peptides (aa 228–239) against the PHF20 Tudor domain. NMR data were processed and analysed with NMRPipe and NMRDraw as previously described^69^. NMR assignments for PHF20 Tudor were taken from the Biological Magnetic Resonance Data Bank (BMRB 17673)^31^.

### Cell culture and RNA interference

Human MCF-7, T-47D, U2OS and HEK293T cells (ATCC) were cultured in DMEM (Cellgro) supplemented with 10% fetal bovine serum (FBS, Sigma). For G9a inhibitor treatment, BIX-01294 (Sigma) was added to the medium, at the indicated concentrations, 24 h before the cells were collected. For shRNA knockdown, 293T cells were co-transfected with pMD2.G and pPAX2 (Addgene) together with pLKO-shRNA constructs or a non-targeting pLKO-shRNA (pLKO-shCtrl). Viral supernatants were harvested after 48 h. For infections, MCF-7 or T-47D cells were incubated with viral supernatants in the presence of 8 μg ml^−1^ polybrene. After 48 h, puromycin (2 μg ml^−1^) was added to the DMEM. Cells were grown, with selection for 5–7 days to select for stable knockdown cells before oestrogen treatment. For oestrogen treatment, cells were starved in phenol red-free DMEM supplemented with 10% charcoal-stripped FBS (Sigma) for 3–4 days, and 10 nM 17β-estradiol (Sigma) or ethanol (vehicle) were added to the medium (3 or 6 h for gene expression studies, and 15 and 45 min for ChIP experiments) before the cells were collected.

### Immunoprecipitation and co-IP

Cell fractionation experiments were performed as described previously^11^. Briefly, cells were collected from one 100-mm dish and resuspended in solution (buffer A) containing 10 mM HEPES pH 7.9, 10 mM KCl, 1.5 mM MgCl_2_, 0.34 M sucrose, 10% glycerol, 1 mM dithiothreitol, and complete protease inhibitor cocktail (Roche). Trition X-100 was added to a final concentration of 0.1%. The cells were incubated for 8 min, and nuclei were collected by centrifugation (1,300*g*, 40 °C, 5 min). The supernatant was clarified by centrifugation (20,000*g*, 40 °C, 5 min) and collected as the cytosolic fraction. The nuclear pellet was washed three times with buffer A and lysed for 30 min in buffer B (3 mM EDTA, 0.2 mM EGTA, 1 mM dithiothreitol and protease inhibitors). The insoluble chromatin fraction and soluble nuclear fractions were separated by centrifugation (1,700*g*, 4 °C, 5 min). The chromatin fraction was washed once with buffer B and sonicated using a Branson digital sonifier. All fractions were boiled in SDS sample buffer and analysed by western blotting.

For immunoprecipitation (IP) experiments, each cell fraction was incubated with the anti-FLAG M2-conjugated agarose beads overnight at 4 °C. The beads were washed 3–6 times with cell lysis buffer, and the bound proteins were eluted in SDS buffer and analysed by western blot. For co-IP experiments, HEK293 cells were transfected with equal amount of vectors expressing Flag- or Myc-tagged proteins as indicated in each Figure. Forty-eight hours after transfection, cells were lysed in cell lysis buffer containing 50 mM Tris-HCl, pH 7.4, 250 mM NaCl, 0.5% Triton X-100, 10% glycerol, 1 mM DTT and a complete protease inhibitor cocktail tablet (Roche), and were treated essentially the same as IP samples forIP and western blot analysis.

### Luciferase reporter assays

U2OS cells were deprived of oestrogen in phenol red-free DMEM supplemented with 10% charcoal-stripped FBS (Sigma) for 1–2 days before transfection. Cells were transfected with 500-ng plasmids of pCAG-ERα (WT or K235R), pCAG-G9a (WT or H1113K), and pCAG-NCOA2, together with 250 ng pERE-Luc-Firefly luciferase and 25 ng pRl-TK-Renilla luciferase plasmids. Forty-eight hours after transfection, the cells were treated with 10 nM 17β-estradiol (Sigma) for 24 h and luciferase activity was measured using the Dual-Luciferase Reporter Assay System (Promega) according to the manufacture's instructions.

### ChIP

ChIP analysis was performed essentially as described previously^11^. Briefly, cells were crosslinked with 1% formaldehyde for 10 min at room temperature, and the reaction was stopped with 125 mM glycine. Nuclei were isolated by resuspending the cells in swelling buffer containing 5 mM PIPES, pH 8.0, 85 mM KCl, 1% NP-40 and complete protease inhibitors for 20 min at 4 °C. The isolated nuclei were resuspended in nuclei lysis buffer (50 mM Tris, pH 8.0, 10 mM EDTA, 1% SDS) and sonicated using a Bioruptor Sonicator (Diagenode). Samples were immunoprecipitated with 2–4 μg of the appropriate antibodies overnight at 4 °C. Immunoprecipitates were washed twice with dialysis buffer (50 mM Tris pH 8.0, 2 mM EDTA, 0.2% Sarkosyl) and four times with IP wash buffer (100 mM Tris, pH 8.0, 500 mM LiCl, 1% NP-40 and 1% deoxycholic acid sodium salt). After reverse crosslinking was performed, the DNA was eluted and purified using a PCR purification kit (Qiagene). The primer sequences used for ChIP analyses are listed in Supplementary Table 4.

### Real-time PCR and RNA-seq analysis

Reverse transcription and real-time PCR were performed as described previously^11^. mRNA was prepared using the RNeasy Plus kit (Qiagen) and reverse transcribed using the First Strand Synthesis kit (Invitrogen). Quantitative real-time RT–PCR (qPCR) was performed on an ABI 7500-FAST Sequence Detection System using the Power SYBR Green PCR Master Mix (Applied Biosystems). Gene expression was calculated following normalization to GAPDH levels using the comparative Ct (cycle threshold) method and is shown as ‘Fold' relative to the expression of each gene in the control cells without E2 treatment that was arbitrarily set as ‘1'. The primer sequences used for RT–PCR are listed in Supplementary Table 5.

RNA-seq samples were sequenced using the Illumina Hiseq 2000, and raw reads were mapped to the human reference genome (hg19) and transcriptome using the RNA-seq unified mapper^70^. Read counts for each transcript were calculated using HTseq v0.6.1 using default parameters^71^. Differential gene expression analyses were performed using the ‘exactTest' function in edgeR v3.0 (ref. 72), with an adjusted *P* value cutoff set to 0.05. For the G9a knockdown data set, the common dispersion was set to 0.04 as suggested in the edgeR manual. For the PHF20 knockdown data set, the common dispersion and tag wise dispersion were calculated using the edgeR ‘estimateCommonDisp' and ‘estimateTagwise-Disp' functions, respectively. The gene expression heatmap was generated using MeV 4.8.1 (ref. 73). Gene Ontology analysis was performed using the DAVID Bioinformatics Resource 6.7 (ref. 74).

### Immunofluorescence staining

MCF-7 cells were E2-depleted for 3 days and treated with 10 nM 17β-estradiol or ethanol for 45 min. Cells were then fixed in 4% formaldehyde, washed and permeabilized in 0.5% Triton X-100 on ice for 10 min. Cells were then blocked in 3% BSA, incubated with primary antibodies in 3% BSA for 2 h, washed, and probed with fluorescein-conjugated secondary antibodies in 3% BSA for 1 h. Cells on the slide were then washed, covered with mounting medium (with DAPI) and observed using an Olympus confocal microscope FluoView FV1000.

### Colony formation assays

MCF-7 cells treated with shRNAs or BIX-01294 were seeded in six-well plates (1,000 cells per well) and grown in DMEM plus 10% FBS at 37 °C for 10–14 days. For anchorage-independent cell growth assays, 4,000 MCF-7 cells were suspended in DMEM containing 0.4% agar and seeded into six-well plates pre-coated with a base layer of 0.6% agar and grown at 37 °C for 4 weeks. Cells were fixed, stained with 0.005% crystal violet blue and photographed. Colony numbers were counted using ImageJ software with size cutoff of 75 μm. Results were quantitated from three to six wells or three to five views of each well from three independent replicates.

## Additional information

**Accession codes:** The RNA-seq data is deposited at GEO repository of National Center for Biotechnology Information under the accession code GSE76507.

**How to cite this article:** Zhang, X. *et al*. G9a-mediated methylation of ERα links the PHF20/MOF histone acetyltransferase complex to hormonal gene expression. *Nat. Commun.* 7:10810 doi: 10.1038/ncomms10810 (2016).

## Supplementary Material

Supplementary Information Supplementary Figures 1-6, Supplementary Tables 1-5

Supplementary Data 1 Lists of E2-activated genes and up- or down-regulated genes in G9a and PHF20 knockdown MCF-7 cells.

Supplementary Data 2 Lists of genes and gene ontology (GO) analysis of G9a-dependent or PHF20-dependent E2 activated genes and the genes that are regulated by both G9a and PHF20.
