# Supplementary material for: G9a-mediated methylation of ERα links the PHF20/MOF histone acetyltransferase complex to hormonal gene expression
Source: Nat Commun. 2016 Mar 10;7:10810. doi: 10.1038/ncomms10810 (PMC4792926; doi:10.1038/ncomms10810)
Supplement: Supplementary Information — Supplementary Figures 1-6, Supplementary Tables 1-5 [file ncomms10810-s1.pdf]

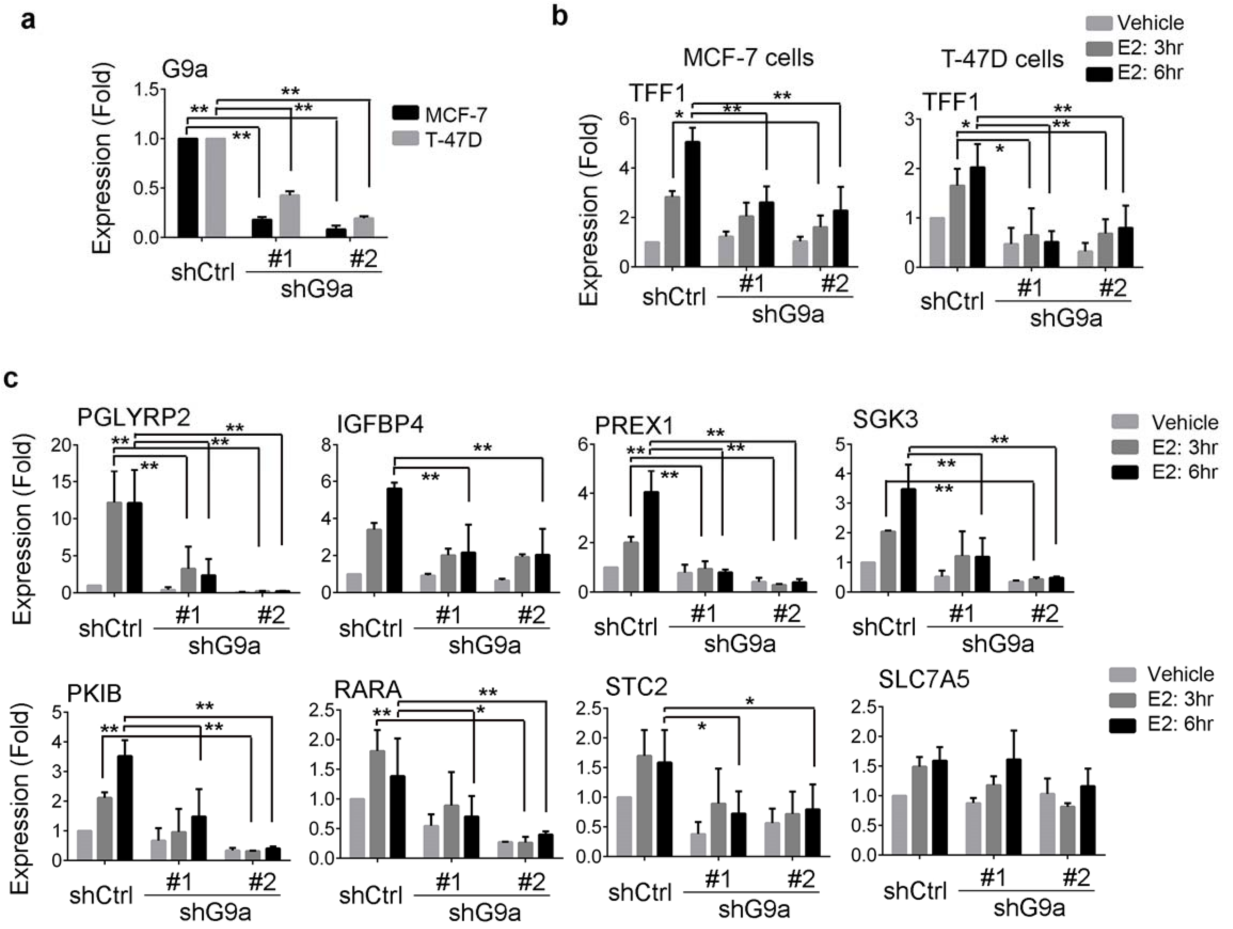

Supplementary Figure 1. G9a is required for the E2-induced expression of endogenous ERα target genes in breast cancer cells.

(**a**) qPCR analysis of G9a mRNA levels in control (shCtrl) and G9a knockdown (shG9a) MCF-7 and T-47D cells. Gene expression was normalized to GAPDH and is shown as fold relative to the expression of each gene in the control cells that was arbitrarily set as "1".

(**b**) G9a is required for E2-induced activation of *TFF1* in MCF-7 (left) and T-47D (right) cells. qPCR analysis of the expression of the *TFF1* gene in control and G9a knockdown cells treated with 10 nM E2 for 3 or 6 hrs.

(**c**) G9a is required for E2-induced activation of a number of ERα target genes in MCF-7 cells. qPCR analysis of the expression of the indicated genes in control and G9a knockdown MCF-7 cells treated with 10 nM E2 for 3 or 6 hrs. *SLC7A5* gene is shown as a negative control.

Gene expression was normalized to GAPDH and is shown as fold relative to the expression of each gene in the untreated control cells. Expression of control genes was arbitrarily set as "1".

All error bars indicate the mean +/- S.E.M. of three experiments. *: $p<0.05$, **: $p<0.01$ (Student's *t*-test).

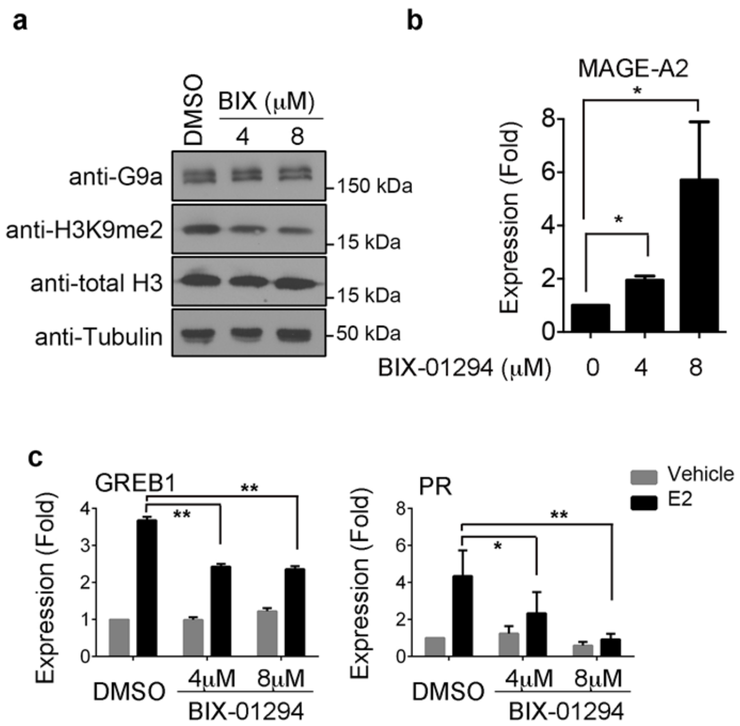

Supplementary Figure 2. BIX-01294 reduces the global H3K9me2 levels and induces the derepression of G9a target genes.

(**a**) BIX-01294 (BIX) reduces global H3K9me2 levels in MCF-7 cells. Western blot analysis showing the H3K9me2 and G9a protein levels in MCF-7 cells treated with 4 or 8 μM BIX-01294. Total H3 and tubulin were used as loading controls.

(**b**) BIX-01294 induces the derepression of G9a-target gene *MAGE-A2*. qPCR analysis of *MAGE-A2* gene expression in cells as in (**a**).

(**c**) BIX-01294 inhibits E2-induced activation of *GREB1* and *PR* in MCF-7 cells. qPCR analysis of gene expression in cells as in (**a**) but treated with 10 nM of E2 for 3 hrs.

In **b, c**, error bars indicate the mean +/- S.E.M. of two experiments. *: $p<0.05$, **: $p<0.01$ (Student's *t*-test).

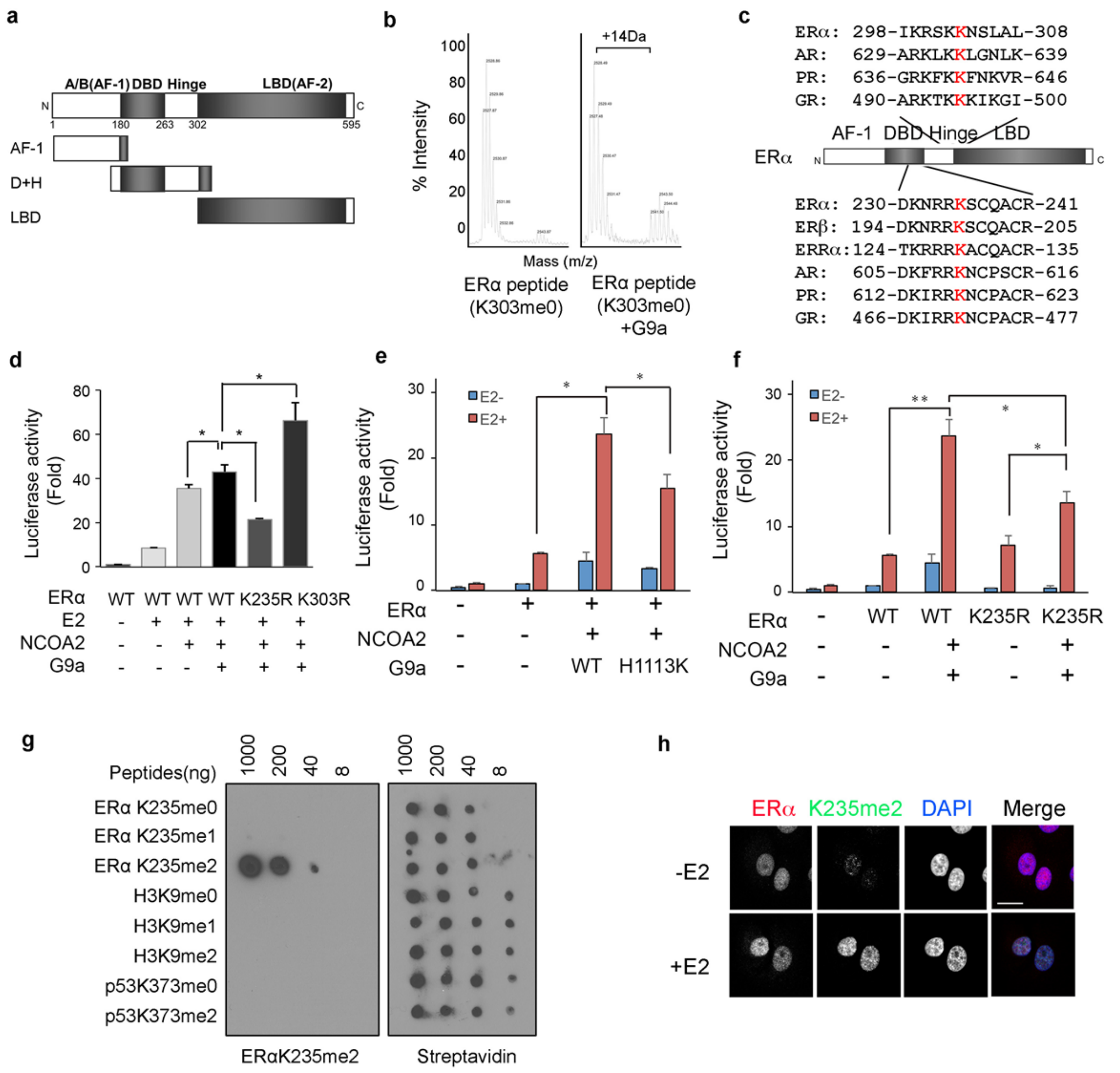

Supplementary Figure 3. G9a methylates ERα and is required for ERα transactivation activity.

(**a**) Schematic representation of the ERα protein domains used in this study. AF-1: activation function 1; DBD: DNA binding domain; H: hinge region; LBD: ligand binding domain. GelCode blue staining shows the input of proteins.

(**b**) G9a monomethylates ERα at K303 *in vitro*. Mass spectrometric analysis of an ERα peptide (aa 294-313) following incubation with or without G9a. The peptide spectra are shown. A change in mass of 14-Daltons indicates the addition of one methyl group.

(**c**) ERαK235 is conserved among nuclear receptors. Alignment of amino acid sequences surrounding K235 (indicated in red, lower alignment) and K303 (indicated in red, upper alignment) in ERα proteins and the indicated nuclear receptors.

(**d**) ERαK235, but not K303, is required for the coactivator function of G9a in an estrogen response element- (ERE) driven luciferase assay. U2OS cells were cotransfected with G9a, NCOA2 and ERα (WT, K235R, or K303R) together with an ERE-Firefly luciferase and a Renilla luciferase, and the luciferase activity was determined 24 hrs after E2 treatment.
(**e**) G9a has a catalytic activity-dependent and independent coactivator activity of ERα. U2OS cells were cotransfected with ERα, NCOA2 and PHF20 (WT or H1113K) together with an ERE-Firefly luciferase and a Renilla luciferase, and the luciferase activity was determined as described in the Method section.
(**f**) Both the E2-dependent and E2-independent ERα coactivator functions of G9a require the ERαK235 residue. U2OS cells were cotransfected with G9a, NCOA2 and ERα (WT or K235R) together with an ERE-Firefly luciferase and a Renilla luciferase, and luciferase activity was determined as described in the Method section.
(**g**) The polyclonal anti-ERαK235me2 antibody is specific. Dot blot analysis of the anti-ERαK235me2 antibody using 5-fold serial dilutions of the indicated biotinylated peptides. HRP-streptavidin was used as a loading control.
(**h**) The ERαK235me2 level is increased upon E2 stimulation. E2-deprived MCF-7 cells were treated with 10 nM 17β-estradiol (+E2) or ethanol (-E2) for 45min, and cells were fixed and immuno-stained using the mouse anti-ERα (1:500) and rabbit anti-ERαK235me2 (1:100) antibodies. DAPI was used to show the DNA content. Scale bar: 20 µm.

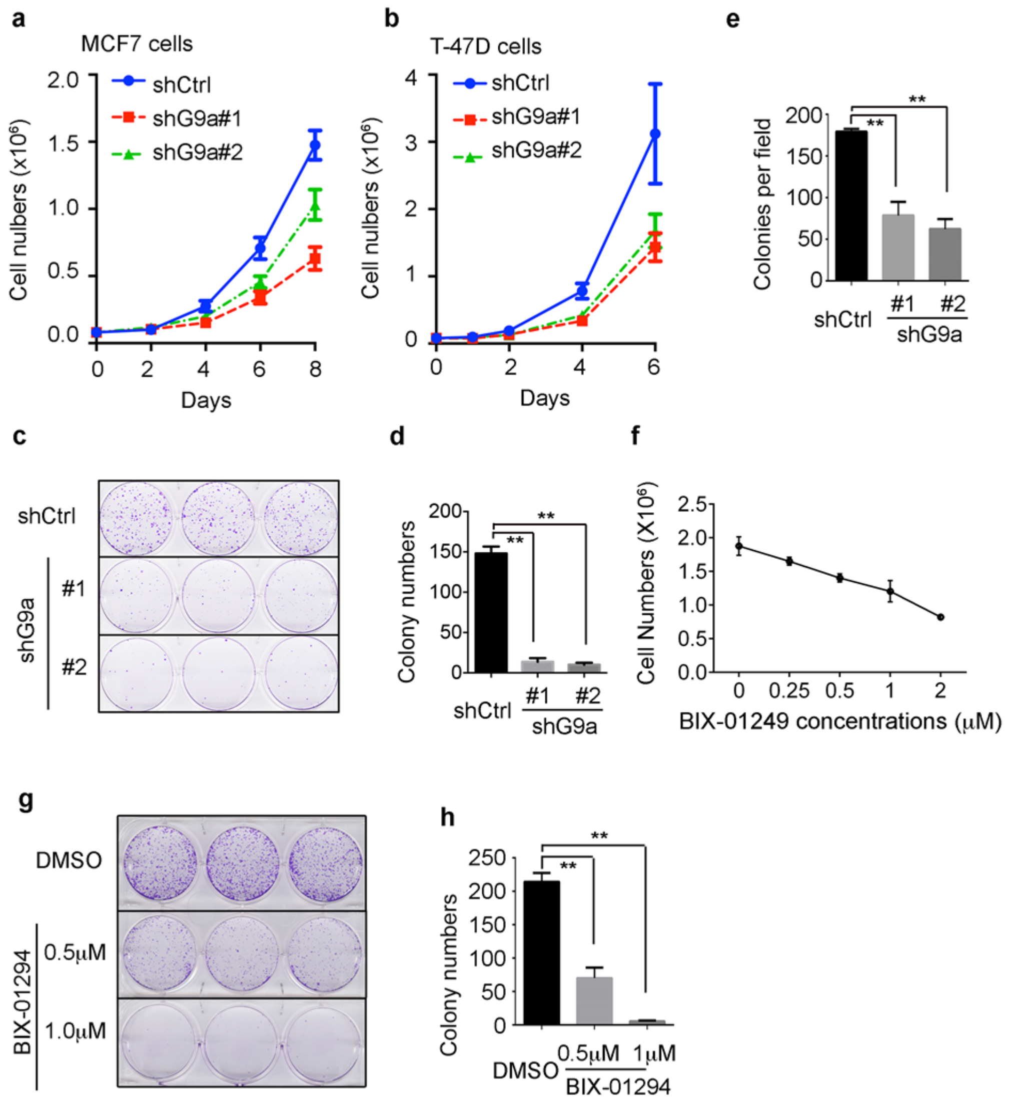

Supplementary Figure 4. G9a is essential for the proliferation of breast cancer cells.
(**a**, **b**) Growth curves of control (shCtrl) and G9a knockdown (shG9a) MCF-7 cells. Cells from 6 wells were counted in triplicates every two days, for 8 days (**a**) or 6 days (**b**) after seeding.
(**c, d**) G9a is required for cell growth in colony formation assays. Representative crystal violet-stained cell colonies are shown (**c**). Colonies arising from control and G9 knockdown MCF-7 cells were counted two weeks after seeding (**d**)
(**e**) G9a depletion reduces the anchorage-independent growth of MCF-7 cells. Cells from control and G9a knockdown MCF-7 were seeded into soft agar. After four weeks, colonies were counted. At least 5 fields of view were counted for each well.
(**f**) The catalytic activity of G9a is required for cell growth. MCF-7 cells treated with the indicated doses of BIX-01249 were counted 6 days after seeding.
(**g, h**) The G9a-selective inhibitor BIX-01249 inhibits cell survival. Representative crystal violet-stained plates are shown (**g**). Colonies arising from cells treated with the indicated doses of BIX-01249 were counted (**h**). All error bars indicate the mean +/- S.E.M. of at least three experiments. **: $p<0.01$ (Student's *t*-test).

Cador 5.0

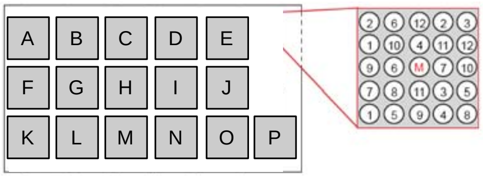

TUDOR<br>A1 TDRD2(NP_006853)<br>A2 TDRD3(Q9H7E2)<br>A3 TDRD4-1(Q9NUY9)<br>A4 TDRD4-2(Q9NUY9)<br>A5 TDRD4-3(Q9NUY9)<br>A6 TDRD7-1(NP_055105)<br>A7 TDRD7-3(NP_055105)<br>A8 EBNA-2Co-A(NP_055205)<br>A9 Ret-bp1(AAB28543)<br>A10 M96(AAH10013)<br>A11 53BP1(1-2)(NP_005648)<br>A12 53BP1(1-2)(NP_005648)	TUDOR +<br>B1 2C(NP_055876)<br>B2 RBP1 like-2 (NP_112739)<br>B3 SMN (NP_075012)<br>B4 PHF20L1(NP_057102)<br>B5 PHF20(NP_057520)<br>B6 PHF20 MBT+TDR(NP_057520)<br>B7 Pombe 1(CAA22823)<br>B8 JMJD2A-2(NP_055478)<br>B9 JMJD2A1-2(NP_055478)<br>B10 LBR TDR(NP_919424)<br>B11 LBR211(NP_919424)<br>B12 SPF30(c)(O75940)	TUDOR / Tudor-Like<br>C1 Lin9 TDR(b)(AAH65302)<br>C2 JMJN 2B WT(f)(NP_055830)<br>C3 ARI4A (e)(NM_002892)<br>C4 PHF19(NP_001009936)<br>C5 SND1 (e)(NM_014390)<br>C6 UHRF1 Tudor-like(a)<br>C7 C420 Tudor-Like(p)<br>C8 C460 Tudor-Like(p)	MBT<br>D1 SFMBT F.L(j)<br>D2 SFMBt 4xMBT(j)<br>D3 L3MBTL1(2-3)(NP_056293)<br>D4 L3MBTL1(1-3)(NP_056293)<br>D5 SCMH1(AAH21252)<br>D6 SCML1(NP_057413)<br>D7 SCML2(AAH64617)<br>D8 LML2(Q969R5)<br>D9 PHF20 MBT(NP_057520)<br>D10 PHF20L1 MBT(NP_057102)	His-MBTs / WD40<br>E1 SFMBT1(a)<br>E2 SFMBT2(a)<br>E3 L3MBTL1(a)<br>E4 L3MBTL2(a)<br>E5 L3MBTL3(a)<br>E6 SCMH1(a)<br>E7 WD40 -WDR5(d)(NP_543124)<br>E8 WD40 -WDR9(NM_018963)<br>E9 WD40 -RbAb46(d)(BT007309)<br>E10 WD40 -RbAb48(d)(X74262)<br>E11 WD40 -HIRA(CR456503)<br>E12 WD40 -Mep50(d)(AF478464)	
PhD +<br>F1 BPTF(P+B)(BAA89208)<br>F2 ING2(e)(AAH50003)<br>F3 PHF2(NP_005383)<br>F4 PHF8(CAI42860)<br>F5 DATF1(CAI95708)<br>F6 Rag2(NM_000536)<br>F7 PCCX1(NP_055408)<br>F8 P300(P+B)(NM_001429)<br>F9 PHF20 PHD(NP_057520)<br>F10 PHD PHF3<br>F11 PHD PHF5<br>F12 PHD CHD5 (1-2)	PhD<br>G1 Dnmt3a-His/GST(g)<br>G2 Dnmt3b-His/GST(g)<br>G3 DnMT3L N-term-His/GST(g)<br>G4 Trim24 Brd-PhD(l)<br>G5 ING3(e) (NP_061944)<br>G6 ING4(e) (NP_001121054)<br>G7 ING5(e) (NP_115705)<br>G8 PHD _TIF1A(e) (O15164)<br>G9 TRI66(e) (O15016)<br>G10 BRPF1(e) (P55201)<br>G11 MLL4(e) (Q9UMN6)<br>G12 MTF2(e) (Q9Y483)	PhD +<br>H1 JMJD2APhD+2Tudor(NP_055478)<br>H2 JMJCPhD(NP_055478)<br>H3 M96Tudor+PhD(AAH10013)<br>H4 MYST4PhD+PhD(AAH48199)<br>H5 NSD1PhD+PWWP(Q96L73)<br>H6 WHSC1PhD+PWWP(NP_579877)<br>H7 BS69PhD+ BRD(AAH12586)<br>H8 ATRX<br>H9 RAL1<br>H10 BAZ1b/WSTF<br>H11 CBP<br>H12 TAF3 (k)	BROMO<br>I1 GCN5(Q92830)<br>I2 TAF1- D1(NP_620278)<br>I3 TAF1- D2(NP_620278)<br>I4 P/CAF (S71788)<br>I5 SNF2 beta(S45252)<br>I6 BAF180 1-2(NP_060635)<br>I7 BAF180-3(NP_060635)<br>I8 BAF180 3-4(NP_060635)<br>I9 BAF180 5-6(NP_060635)<br>I10 KAP-1(AAB37341)<br>I11 P300(NP_004371)<br>I12 WDR9 2(Q9NSI6)	BROMO /SANT / TSN<br>J1 Bromo-BAZ(NP_075381)<br>J2 Bromo-BRDT 1(AAH62700)<br>J3 SANT-MPP11-like(XP_379909)<br>J4 SANT-N-CoR2-2(Q9Y618)<br>J5 SANT-RERE(AAH62342)<br>J6 SANT-ADA2(NP_001479)<br>J7 SANT-Zuotin Rel.(XP_168590)<br>J8 TSN-p100(o) (NP_055205)<br>J9 TSN-p100 m5(o) (NP_055205)<br>J10 TSN-p100 m6(o) (NP_055205)	
CHROMO<br>K1 TIP60(h)(AAB18236)<br>K2 CHD2(h)(AAB87382)<br>K3 CHD4(h)(AAH38596)<br>K4 MPP8(h) (NP_059990)<br>K5 SMARCC2(h)(AAH26222)<br>K6 MRG15(h)(AAD29872)<br>K7 RBBP1(h)(AAD41239)<br>K8 PC2(h)(AAB80718)<br>K9 PC3(h)(AAG09180)<br>K10 CHD5(h)(AAK56405)<br>K11 CHD7 (1-2)(AB037837)<br>K12 CBX6/NPCD(BC012111)	CHROMO<br>L1 MI-2(h)(CAA60384)<br>L2 HP1alpha(h)(P45973)<br>L3 HP1gamma(h)(NP_057671)<br>L4 MSL3-like(h)(AAD38499)<br>L5 SUV39H1(h)(AAB92224)<br>L6 CBX1/HP1beta(h)(AAD21972)<br>L7 HP1 beta(h)(P23197)<br>L8 CDY1(h)(AAD22735)<br>L9 CHD1(e) (NP_001261)<br>L10 CBX4/PC2(e) (NM_003655)<br>L11 CBX7/RP4(e) (NM_175709)<br>L12 CBX5/HP1alpha(e)NM_012117)	CHROMO / BRK /MRG<br>M1 CBX3/HP1gamma(e)(NM_016587)<br>M2 CBX2/CDCA6(e) (NM_005189)<br>M3 CDYL2(e) (NM_152342)<br>M4 CBX8/PC3(e) (NM_020649)<br>M5 BRK_SMCA2 (e)(NM_003070)<br>M6 BRK_SMCA4(e) (NM_003072)<br>M7 BRK_CHD6(e) (NM_032221)<br>M8 BRK_CHD7 (e)(NM_017780)<br>M9 BRK_Q6DTK9 (e)(NM_025134)<br>M10 MRG_MSL3v1(e) (NM_078629)<br>M11 MRG15(e) (NM_206839)	PWWP<br>N1 BRPF1(AAH53851)<br>N2 DNMT3B(Q9UBC3)<br>N3 HDGF (P51858)<br>N4 HRP-3(BAA90477)<br>N5 MSH6(P52701)<br>N6 NSD1(Q96L73)<br>N7 WHSC1-1(NP_579877)<br>N8 PSIP1(e) (NM_033222<br>N9 BRD1(e) (NM_014577<br>N10 ZCPW1 (e)(AL136735<br>N11 MBD5 (e)(NM_018328	PWWP / CW / SWIRM<br>O1 PWWP_PKCB1(e) (NM_183047<br>O2 PWWP_HDGR3(e) (NM_016073)<br>O3 PWWP_DNM3A (e) (NM_175629)<br>O4 CW3(AAH02725)<br>O5 CW5(BAA09485)<br>O6 CW6(XP_087384)<br>O7 SWIRM_ KIAA1915(BAB67808)<br>O8 SWIRM_KIAA0601(CAB72299)<br>O9 SWIRM_ADA2(NP_001479)	ANK<br>Q1 ANK-BARD1 (NP_000456)<br>Q2 ANK-GLP(m) (AAM09024.1)<br>Q3 ANK-Notch (NP_060087)<br>Q4 ANK-IKB alpha F.L. (e)<br>Other<br>Q5 TULP1<br>Q6 MeCP2(i)<br>Q7 PHD_ ZFP-1 His/GST(n)

Recombinat Proteins Construct Sources

a - Cheryl Arrowsmith	d - Jiemin Wong	g - Albert Jeltsch	j - Judd Rice	n - Alla Grishok
b - Subhashini Sadasivem	e - Or Gozani	h - Yi Zhang	k - Marc Timmers	o - Olli Silvennoinen
c - Jocelyn Coté	f - Thomas Jenuwein	i - Adrian Bird	l - Michelle Barton	p - Logan Donaldson
			m- Michael Stallcup	

Supplementary Figure 5. Domain layout of the Chromatin-Associated Domain Array (CADOR 5.0).

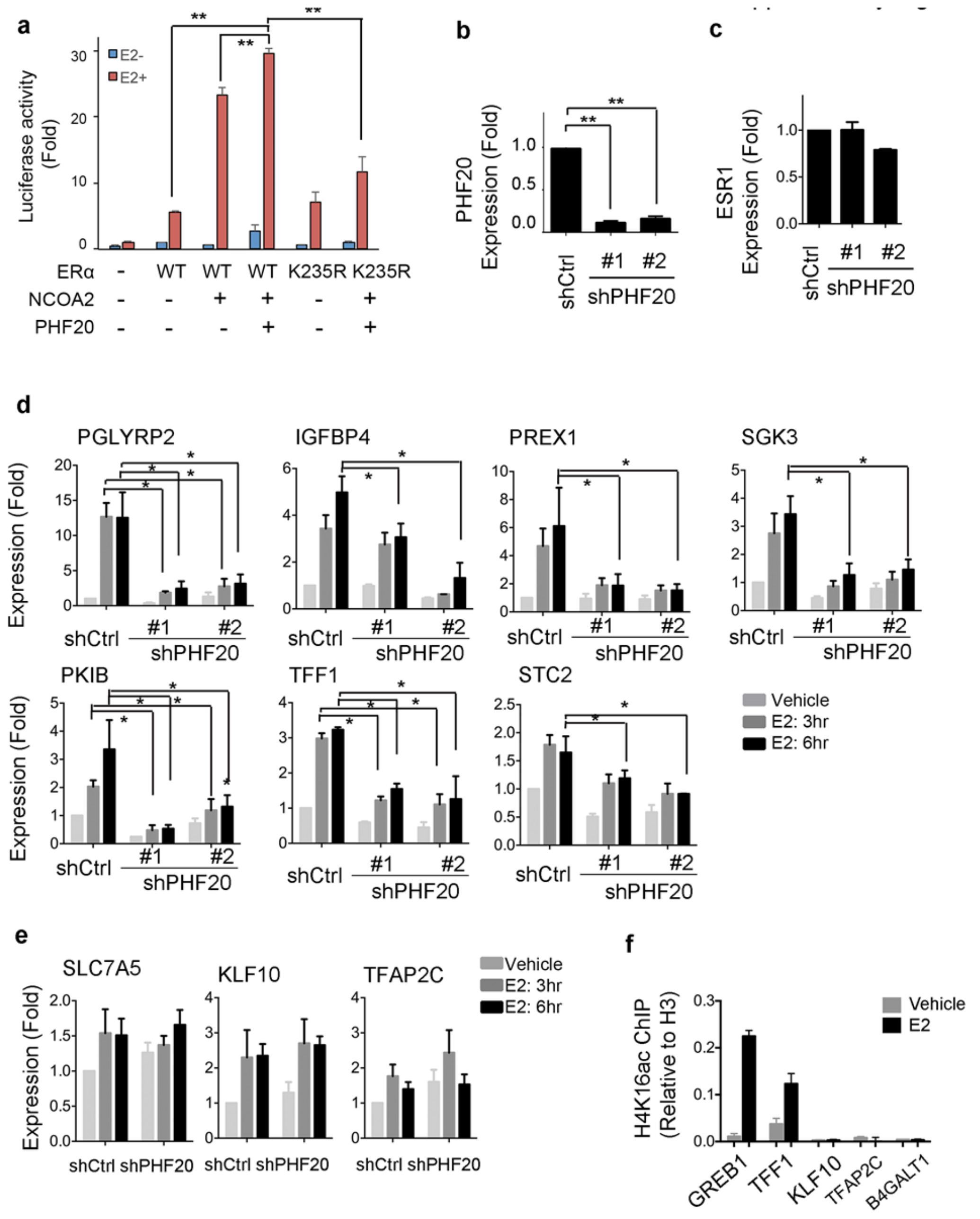

Supplementary Figure 6. PHF20 is required for the E2-induced expression of endogenous ERα target genes.

(**a**) The ERα coactivator function of PHF20 requires the ERαK235 residue. U2OS cells were cotransfected with PHF20, NCOA2 and ERα (WT or K235R) together with an ERE-Firefly luciferase and a Renilla luciferase, and the luciferase activity was determined 24 hrs after E2 treatment.

(**b, c**) qPCR analysis of PHF20 (**b**) and ERα (**c**) mRNA levels in control (shCtrl) and PHF20 knockdown (shPHF20) MCF-7 cells.

(**d, e**) PHF20 is required for E2-induced activation of a number of ERα target genes in MCF-7 cells. qPCR analysis of the expression of the indicated genes in control and PHF20 knockdown

MCF-7 cells treated with 10 nM E2 for 3 or 6 hrs. *SLC7A5, KLF10* and *TFAP2C* genes were used as controls for PHF20-indepdent genes (**e**).
(**f**) H4K16ac is enriched on PHF20-dependent but not PHF20-independent ERα target genes. qPCR analysis of histone H4K16ac ChIP on the promoters of the PHF20-dependent genes (*GREB1* and *TFF1)* and PHF20-indepdent genes (*KLF10, TFAP2C* and *B4GALT1*) in MCF-7 cells +/- E2 treatment.
In panels **b-e**, all gene expression data is shown as the fold increase or decrease relative to the expression of each gene in the untreated control cells. Control gene expression was arbitrarily set as "1". All error bars indicate the mean +/- S.E.M. of three independent experiments. *: $p<0.05$, **: $p<0.01$ (Student's *t*-test).

Supplementary Table 1. Gene ontology (GO) analysis of G9a-dependent E2-activated genes (group A) in MCF-7 cells. The top ten enriched categories are shown (*p*<0.05, Fisher's exact test). The entire list of GO terms is shown in Supplementary Data 2.

Gene Ontology Term	Count	*P*-Value
Morphogenesis of an epithelium	8	7.0E-04
Tissue morphogenesis	10	1.3E-03
Epithelium development	11	1.9E-03
Intracellular signaling cascade	32	2.6E-03
Metabotropic glutamate receptor signaling pathway	3	4.3E-03
Cell surface receptor linked signal transduction	40	1.2E-02
Germ cell migration	3	1.3E-02
Epithelial tube morphogenesis	5	1.7E-02
Positive regulation of protein kinase activity	9	1.7E-02
Neuroprotection	3	1.7E-02

Supplementary Table 2. GO analysis of PHF20-depedent E2-activated genes (253 genes) in MCF-7 cells. The entire list of GO terms is shown in Supplementary Data 2.

**Gene Ontology Term**	**Count**	***P*-Value**
Regulation of DNA binding	7	0.003
Regulation of binding	7	0.008
Regulation of system process	10	0.009
Cell adhesion	16	0.014
Biological adhesion	16	0.014
Tube morphogenesis	6	0.015
Intracellular signaling cascade	24	0.015
Epithelium development	8	0.015
Response to hypoxia	6	0.019
Protein amino acid phosphorylation	15	0.020

Supplementary Table 3. GO analysis of the 159 E2-activated genes that require both G9a and PHF20 for activation in MCF-7 cells. The entire list of GO terms is shown in Supplementary Data 2.

Gene Ontology Term	Count	*P*-Value
Epithelium development	7	0.007
Monovalent inorganic cation transport	8	0.009
Epithelial tube morphogenesis	4	0.013
Tube morphogenesis	5	0.015
Tube development	6	0.024
Protein amino acid autophosphorylation	4	0.025
Peptidyl-citrulline biosynthetic process from peptidyl-arginine	2	0.036
Morphogenesis of an epithelium	4	0.039
Embryonic epithelial tube formation	3	0.040
Tube lumen formation	3	0.042

Supplementary Table 4. Sequences of ChIP primers used in this study

Primer	Sequence
GREB1-distal-ERE-F	TCACCGATGTTCACATAGCTAATTC
GREB1-distal-ERE-R	CCCGAAGCTGAACACTCTTTG
GREB1-proximal-ERE-F	GCCTGAAGTGACCAGCTTTTTG
GREB1-proximal-ERE-R	GCAGGTGCTCGCTTGCA
PR-distal-ERE-F	AATTTTGCAATGGCTCTGCAT
PR-distal-ERE-R	CGGATCTCCTGGAAAATGTCA
PR-proximal-ERE-F	TTGGTTCTGCTTCGGAATCTG
PR-proximal-ERE-R	CCTCCTCTCCTCACTCTTGG
TFF1-distal-ERE-F	CTGGGTGACAGGAAAGAAGC
TFF1-distal-ERE-R	CATTCTGGAAGGGACACACA
TFF1-proximal-ERE-F	GCTTAGGCCTAGACGGAATGGGC
TFF1-proximal-ERE-R	CCAGGTCCTACTCATATCTGAGAG
KLF10-proximal-F	GAGGATAGCTTGAGCCCAGAAG
KLF10-proximal-R	AGGGATGAAAAAAAACTGCTCTGT
TFAP2C-proximal-F	CCAGGTGACATCCGTCTTTTG
TFAP2C-proximal-R	CCACCCCTCTTTCGCCTAA
B4GALT1-proximal-F	TTCCACGGCCACCAAATT
B4GALT1-proximal-R	CGCAGTGTCACCCCAGAGT

Supplementary Table 5. Sequences of RT-PCR primers used in this study

Primer	Sequence
GREB1-RT-1F	GGAAAATACCAAGCCCGGATT
GREB1-RT-1R	TCGTAATTGACTTCCTTCTGGTACTC
PR-RT-1F	GGAGATGAGGTCAAGCTACATTAGAGA
PR-RT-1R	CGCTGTGAGCTCGACACAAC
TFF1-RT-1F	TTTCGACGACACCGTTCGT
TFF1-RT-1R	GGAGGGACGTCGATGGTATTAG
G9a-RT-1F	TCCGCTGATTTTCGAGTGTAAC
G9a-RT-1R	CCCGGTTCTTGCAGTTTCTC
PGLYRP2-RT-1F	CAGGGTTCCGCAGCAACT
PGLYRP2-RT-1R	CCAGGATGGAGGCTGAAGTC
IGFBP4-RT-1F	TGGACTGAATGTGCCTAATGGA
IGFBP4-RT-1R	CCCAGGAAGCCCCTCATC
PREX1-RT-1F	ATCCACCATGTTTCCGACAAG
PREX1-RT-1R	TCGTAGCGGAAGCGATACATC
SGK3-RT1-F	TCTGGAAATCCTCATGCCAAA
SGK3-RT1-R	CAAAGCTGCCTTTTCCAATAACTT
PKIB-RT-1F	TCGCCAATTTTGCATCTTCA
PKIB-RT-1R	TGGATGTCTGGTAAGGCATTCC
RARA-RT-1F	GCCTCTCATCCAGGAAATGTTG
RARA-RT-1R	CGGCTGTCCGCTCAGAGT
STC2-RT-1F	CGGCTGCCCAGGAGAAC
STC2-RT-1R	GCAAGTCCTTGAAATGGATCATC
SLC7A5-RT-1F	GGTGTACGTGCTGACCAACCT
SLC7A5-RT-1R	CCTCGGACGACAGCATCTG
PHF20 RT-1F	TCGAAGCCTGCCATAAAGGTA
PHF20 RT-1R	TTCATTCAACGATGTGCACAGA
ESR1-RT-1F	TCTGCCAAGGAGACTCGCTACT
ESR1-RT-1R	CACAGGACCAGACTCCATAATGG
KLF10 RT-F	AAAGCTCAGGCAACAAGTGTGA
KLF10 RT-R	TTGGGCAGGTCTGGTGGTTA
TFAP2C RT-F	ATCTTGGAGGACGAAATGAGATG
TFAP2C RT-R	TGCTGGGCCGCCAATA
MAGE-A2 RT-F	CCAAGACAGGCCTCCTGATAAT
MAGE-A2 RT-R	GGCACAGTCGCCCTCTATTG
